# Original research: initial development of a pragmatic tool to estimate cognitive decline risk focusing on potentially modifiable factors in Parkinson’s disease

**DOI:** 10.3389/fnins.2023.1278817

**Published:** 2023-10-24

**Authors:** Tara C. Carlisle, Luis D. Medina, Samantha K. Holden

**Affiliations:** ^1^Department of Neurology, Behavioral Neurology Section, University of Colorado School of Medicine, Aurora, CO, United States; ^2^University of Colorado Alzheimer’s and Cognition Center, Aurora, CO, United States; ^3^University of Colorado Movement Disorders Center, Aurora, CO, United States; ^4^Department of Psychology, University of Houston, Houston, TX, United States; ^5^Department of Neurology, Movement Disorders Section, University of Colorado School of Medicine, Aurora, CO, United States

**Keywords:** Parkinson disease, cognitive dysfunction, dementia, modifiable risk factors, predictor tool

## Abstract

**Introduction:**

Cognitive decline is common in Parkinson’s disease (PD). Calculating personalized risk of cognitive decline in PD would allow for appropriate counseling, early intervention with available treatments, and inclusion in disease-modifying trials.

**Methods:**

Data were from the Parkinson’s Progression Markers Initiative *de novo* cohort. Baseline scores were calculated for Lifestyle for Brain Health (LIBRA) and the Montreal Parkinson Risk of Dementia Scale (MoPaRDS) per prior literature and preliminary Parkinson’s disease Risk Estimator for Decline In Cognition Tool (pPREDICT) by attributing a point for fourteen posited risk factors. Baseline and 5-year follow-up composite cognitive scores (CCSs) were calculated from a neuropsychological battery and used to define cognitive decliners (PD-decline) versus maintainers (PD-maintain).

**Results:**

The PD-decline group (*n* = 44) had higher LIBRA (6.76 ± 0.57, *p* < 0.05), MoPaRDS (2.45 ± 1.41, *p* < 0.05) and pPREDICT (4.52 ± 1.66, *p* < 0.05) scores compared to the PD-maintain group (*n* = 263; LIBRA 4.98 ± 0.20, MoPaRDS 1.68 ± 1.16, pPREDICT 3.38 ± 1.69). Area-under-the-curve (AUC) for LIBRA was 0.64 (95% confidence interval [CI], 0.55–0.73), MoPaRDS was 0.66 (95% CI, 0.58–0.75) and for pPREDICT was 0.68 (95% CI, 0.61–0.76). In linear regression analyses, LIBRA (*p* < 0.05), MoPaRDS (*p* < 0.05) and pPREDICT (*p* < 0.05) predicted change in CCS. Only age stratified by sex (*p* < 0.05) contributed significantly to the model for LIBRA. Age and presence of hallucinations (*p* < 0.05) contributed significantly to the model for MoPaRDS. Male sex, older age, excessive daytime sleepiness, and moderate–severe motor symptoms (all *p* < 0.05) contributed significantly to the model for pPREDICT.

**Conclusion:**

Although MoPaRDS is a PD-specific tool for predicting cognitive decline relying on only clinical features, it does not focus on potentially modifiable risk factors. LIBRA does focus on potentially modifiable risk factors and is associated with prediction of all-cause dementia in some populations, but pPREDICT potentially demonstrates improved performance in cognitive decline risk calculation in individuals with PD and may identify actionable risk factors. As pPREDICT incorporates multiple potentially modifiable risk factors that can be obtained easily in the clinical setting, it is a first step in developing an easily assessable tool for a personalized approach to reduce dementia risk in people with PD.

## 1. Introduction

About 10–20% of individuals newly diagnosed with Parkinson’s disease (PD) have cognitive changes (Aarsland et al., 2009; Weintraub et al., 2015) and cognitive decline is associated with high rates of conversion to dementia (Aarsland et al., 2003). Multiple tools have been developed to predict risk of cognitive decline in PD (Schrag et al., 2017; Dawson et al., 2018; Hogue et al., 2018; Gramotnev et al., 2019; Yousaf et al., 2019; Wilson et al., 2020), however these tools rely on testing not routinely performed outside of tertiary care or research settings (e.g., dopamine transporter (DaT) imaging (Schrag et al., 2017; Yousaf et al., 2019), formal olfactory testing (Schrag et al., 2017), cerebral spinal fluid (CSF) studies (Schrag et al., 2017; Gramotnev et al., 2019; Yousaf et al., 2019), and genetic testing (Gramotnev et al., 2019). There are tools using specific neuropsychological assessments (Hogue et al., 2018; Wilson et al., 2020); however, the selection of formal neuropsychological tests is not standardized nor are all formal neuropsychological tests available in all clinical settings. The Montreal Parkinson Risk of Dementia Scale (MoPaRDS) is an office-based tool for estimating risk of developing dementia in PD and therefore holds promise for scalability to a wider PD population (Dawson et al., 2018); however, MoPaRDS does not focus on potentially modifiable risk factors allowing for actionable lifestyle or treatment changes based on personalized risk assessment.

Indeed, although previous PD-specific predictor tools, including the MoPaRDS, provide information about the risk of cognitive decline (Schrag et al., 2017; Dawson et al., 2018; Hogue et al., 2018; Gramotnev et al., 2019; Yousaf et al., 2019; Wilson et al., 2020), none focus on modifiable risk factors. To our knowledge, there is currently no PD-specific predictor tool for cognitive decline that focuses on risk factors that have potential interventions for immediate action with lifestyle modifications or available symptom-based treatments. The Lifestyle for Brain Health (LIBRA) score focuses on modifiable, lifestyle-related risk factors associated with all-cause dementia identified through systematic literature review and Delphi consensus (Deckers et al., 2015; Vos et al., 2017; Schiepers et al., 2018). Components of the full LIBRA score can be easily assessed in routine clinical settings and include demographics and modifiable risk factors (Table 1; Deckers et al., 2015). To our knowledge, LIBRA has not been validated to assess risk of cognitive decline specifically in a population of individuals with PD.

**Table 1:** LIBRA, MoPaRDS, and pPREDICT Risk Factors and Scoring Systems

**LIBRA**		**MoPaRDS** (Dawson et al., 2018)	**pPREDICT**
*Demographic*	Points	*Demographic*	*Demographic*
Age for males/females		Older age (≥70 years)	Older age [≥65 years (Pedersen et al., 2017)] (Baiano et al., 2020; Guo et al., 2021)
<65 years	0	Male sex	Male sex (Reekes et al., 2020)
65-69 years	0.4 / 2.1		Non-white race and/or Hispanic ethnicity (Ben-Joseph et al., 2020)
70-74	5.2 / 6.2		
75-79	6.8 / 9.2		
80-84	11.2 / 12.4		
85-89	14.1 / 15.3		
≥90	16.4 / 17.6		
Educational level			Lower educational attainment [≤12 years (Segura et al., 2014; Mak et al., 2015; Santangelo et al., 2015; Peraza et al., 2017)] (Baiano et al., 2020)
High [≥13 years (van Middelaar et al., 2018)]	0		
Medium (van Middelaar et al., 2018)	1.4		
Low [≤7 years (van Middelaar et al., 2018)]	2.7	*Clinical*	*Clinical*
		Mild Cognitive Impairment (MoCA <26)	Lower MoCA score [<26 (Nasreddine et al., 2005)] (Kim et al., 2019)
		Orthostatic hypotension (SBP drop >10 mmHg)
		*PD-specific*	*PD-specific*
		Bilateral disease onset*	Longer disease duration [≥5 years (Hong et al., 2018; Li et al., 2019)] (Baiano et al., 2020)
		Falls/freezing (UPDRS 2.12 >1 and/or 2.13 >0)	
		*PD-specific – potentially modifiable or treatable*	*PD-specific – potentially modifiable or treatable*
		RSBD (RSBDQ >5)	RSBD [RSBDQ ≥5 (Chahine et al., 2013)] (Forbes et al., 2021; Guo et al., 2021; Xie et al., 2021)
		Visual hallucinations (UPDRS 1.2 >0)	Moderate-severe motor [UPDRS Pt. III ≥32 (Martinez-Martin et al., 2015)] (Riggeal et al., 2007; Guo et al., 2021)
*Potentially modifiable or treatable*			*Potentially modifiable/treatable*
Depression [GDS-15 ≥5 (van Middelaar et al., 2018)]	2.1		Depression [GDS-15 ≥8 (Dissanayaka et al., 2007)] (Forbes et al., 2021)
			Anxiety [STAI-S ≥54 (Julian, 2011)] (Guo et al., 2021)
			Excessive daytime sleepiness [ESS ≥11 (Johns, 1991)] (Forbes et al., 2021)
Obesity [BMI ≥30 kg/m^2^ (Deckers et al., 2018; van Middelaar et al., 2018)]	1.6		Obesity (BMI ≥25 kg/m^2^) (Forbes et al., 2021)
Physical inactivity [PASE >90 (Deckers et al., 2015)]	1.1		Low physical activity [PASE ≤90 (Curcio et al., 2019)] (Paul et al., 2019)
Vascular risks			Significant vascular risk factors [≥2 of CAD, ischemic stroke/TIA, HLD, HTN and/or DM (Gottesman et al., 2017)] (Pilotto et al., 2016; Gottesman et al., 2017; Chahine et al., 2019; Nicoletti et al., 2021)
Hypertension	1.6	
Hypercholesterolemia	1.4	
Diabetes	1.3		
Coronary heart disease	1.0	**All MoPaRDS risk factors are worth 1 point.**	**All pPREDICT risk factors are worth 1 point.**

For LIBRA, references to determine dichotomous cut-off values [blue] are provided. For MoPaRDS, single reference used for all risk factors and cut-off values. For pPREDICT, both references to identify risk factors associated with cognitive decline in PD [black] and references to specifically determine dichotomous cut-off values [blue] are provided.

Full LIBRA index also contains smoking status, alcohol use history, diet, and cognitively based activities, however these risk factors were not included in this analysis due to missingness of data in the PPMI database.

*For bilateral disease onset determination, scores from Unified Parkinson’s Disease Rating Scale (UPDRS) part III questions 3.3-3.8 were tallied comparing right from left; bilateral onset was designated if the difference between the total scores for right and left was <3.

***Abbreviations***: BMI = body mass index; CAD = coronary heart disease; ESS = Epworth Sleepiness Scale; GDS-15 = Geriatric Depression Scale Short Form; HLD = hyperlipidemia; HTN = hypertension; LIBRA = Lifestyle for Brain Health; MoCA = Montreal Cognitive Assessment; PASE = Physical Activity Scale for the Elderly; pPREDICT = preliminary Parkinson’s disease Risk Estimator for Decline In Cognition Tool; UPDRS = Unified Parkinson’s Disease Rating Scale; RSBDQ = REM Sleep Behavior Disorder (RSBD) Questionnaire; SBP = systolic blood pressure; STAI-S = State-Trait Anxiety Inventory (State); TIA = transient ischemic attack.

The goal of this study was to explore development of a pragmatic tool to estimate personal risk of cognitive decline specifically for people with PD while focusing on modifiable factors. LIBRA was used as a starting point given its utility in all-cause dementia prediction, but additional risk factors easily obtained in routine clinical assessment without expensive or invasive testing or procedures were identified to explore a PD-specific tool. MoPaRDS was used as a PD-specific comparator tool given its utility in the clinical setting even outside of tertiary/quaternary care centers or in the research setting. The fourteen putative, dichotomous risk factors for the preliminary Parkinson’s disease Risk Estimator for Decline In Cognition Tool (pPREDICT) included demographic, clinical, PD-specific, and potentially reversible and/or treatable risk factors based on literature review (Table 1; Riggeal et al., 2007; Pilotto et al., 2016; Gottesman et al., 2017; Chahine et al., 2019; Kim et al., 2019; Paul et al., 2019; Baiano et al., 2020; Ben-Joseph et al., 2020; Reekes et al., 2020; Forbes et al., 2021; Guo et al., 2021; Nicoletti et al., 2021; Xie et al., 2021). There are no disease-modifying therapeutics to slow the cognitive decline associated with PD, therefore focusing on potentially reversible and/or treatable risks is of particular importance. Additionally, focusing on easily obtained clinical markers allows for greater accessibility to the wider PD population, which is of upmost importance given that many (40%) individuals with PD do not see a neurologist every year and even fewer (1.8%) are treated by movement disorder specialists (Pearson et al., 2023). A predictor tool focusing on lifestyle-related and/or treatable PD-specific risk factors opens the door for individualized treatment plans to slow or prevent cognitive decline in PD.

## 2. Methods

### 2.1. Study population and data handling

Data were obtained from the Parkinson’s Progression Markers Initiative (PPMI) database.^1^ For up-to-date information on the study, visit www.ppmi-info.org. Data were downloaded on September 2, 2019 with updated neuropsychological data downloaded June 7, 2021. The study was approved by the Colorado Multiple Institutional Review Board (exempt protocol #20–3173).

Participants from the *de novo* (i.e., recently diagnosed) cohort with PD containing baseline and 5-year follow-up neuropsychological testing scores were included in the final analysis (*n* = 307). Participants defined as PD without available DaT imaging and scans without evidence of dopaminergic deficit (SWEDD) were not included to reduce the heterogeneity of the participant sample. There were missing data for disease duration and race/ethnicity for two participants and therefore not included in the linear regression analyses for pPREDICT (*n* = 305).

### 2.2. Neuropsychological battery and composite cognitive score

Five neuropsychological measures spanning different cognitive domains were included in the analysis: (1) attention and working memory: WAIS-IV Letter Number Sequencing (LNS) (Wechsler, 1987), (2) executive function: Symbol Digit Modalities Test (SDMT) (Smith, 1973), (3) language: category/semantic (animal) fluency (SF) (Straus et al., 2006), (4) memory: Hopkins Verbal Learning Test, Delayed Recall (HVLT-DR) (Brandt, 1991), and (5) visuospatial function: Benton’s Judgment of Line Orientation (JLO) (Benton et al., 1978). Raw neuropsychological test scores for JLO, SDMT, and SF were converted to z-scores based on normative data drawn either from testing manuals (Smith, 1973; Benton et al., 1978) or additional studies (Tombaugh et al., 1999). For HVLT-DR and LNS, the PPMI-derived T-scores and scaled scores, respectively, were converted to z-scores. Baseline and 5-year follow-up composite cognitive scores (CCS) were calculated as a mean of the z-scores. CCS are routinely used in Alzheimer’s disease research (Langbaum et al., 2014; Burnham et al., 2015) and used in PD cognitive research (Wood et al., 2021).

The Montreal Cognitive Assessment (MoCA) is a sensitive screening tool for cognitive impairment in PD (Chou et al., 2010; Kim et al., 2019), however is not intended to replace formal neuropsychological testing. The MoCA is a screening tool that identifies individuals with PD at higher risk for cognitive impairment, but even with validated cut-off values does not perform perfectly with only 64% of individuals with PD correctly diagnosed on the cognitive spectrum (Hoops et al., 2009); therefore, it is not unreasonable to use as a risk factor for cognitive decline. The MoCA score was also not used for the outcome measure to determine cognitive decline (CCS), thereby avoiding circularity in the statistical analysis for MoPaRDS and pPREDICT. Additionally, MoCA is a cognitive screen that can easily be performed in multiple clinical settings (Nasreddine et al., 2005), therefore use is not restricted to tertiary/quaternary care centers or in the research setting making it ideal for the goal of wider access.

### 2.3. Cognitive trajectory classification

Cognitive decliners (PD-decline) were defined as ≥0.5 standard deviation (SD) CCS decline from baseline to 5-year follow-up and cognitive maintainers (PD-maintain) as <0.5 CCS decline. This cutoff was chosen given Movement Disorder Society Task Force Guidelines for diagnosing PD mild cognitive impairment (PD-MCI) based on significant decline on serial neuropsychological testing (Litvan et al., 2012); since PPMI is a highly educated cohort (Marek et al., 2018), significant decline over a 5-year period was defined as ≥0.5 SD CCS.

### 2.4. LIBRA scoring

For LIBRA score calculations, points were attributed to each risk factor as previously described (Vos et al., 2017; Table 1). Smoking status, alcohol use history, diet, and cognitively based activities are not reliably available through PPMI due to missingness of data, but there is precedence for omitting components of LIBRA due to absence of data in establish cohorts (Vos et al., 2017; van Middelaar et al., 2018). Risk factors from PPMI data requiring specific cutoff values for LIBRA were based on previous studies applying the LIBRA score (Table 1; Deckers et al., 2018; van Middelaar et al., 2018). We were unable to identify any prior publications using Physical Activity for the Elderly Scale (PASE) for calculating LIBRA. However, original Delphi consensus compared highest to lowest level of activity (Deckers et al., 2015); therefore, a cutoff for moderate-intense activity (i.e., PASE >90) was selected.

### 2.5. MoPaRDS scoring

For MoPaRDS score calculations, a point was added for each of the eight risk factors as previously described (Dawson et al., 2018; Table 1). As the PPMI database was used previously for MoPaRDS, the same cutoff values were used (Dawson et al., 2018).

### 2.6. pPREDICT scoring

For pPREDICT, dichotomous cutoff values for risk factors were chosen based on literature search (Table 1; Johns, 1991; Nasreddine et al., 2005; Dissanayaka et al., 2007; Julian, 2011; Chahine et al., 2013; Segura et al., 2014; Mak et al., 2015; Martinez-Martin et al., 2015; Santangelo et al., 2015; Gottesman et al., 2017; Pedersen et al., 2017; Peraza et al., 2017; Hong et al., 2018; Curcio et al., 2019; Li et al., 2019; Ben-Joseph et al., 2020; Reekes et al., 2020; Forbes et al., 2021). For total pPREDICT score calculations, the predicted dichotomous risk factors each represented a point toward fourteen possible total points. This point system is similar to other screening tools designed for easy use in the clinical setting, including the office-based MoPaRDS screen for cognitive decline in PD that showed no significant improvement in diagnostic accuracy with weighting (Dawson et al., 2018).

### 2.7. Statistical analysis

Clinical and demographic features of the PD-decline and PD-maintain groups were evaluated using descriptive statistics. Groupwise comparisons were made using either t-tests or Mann Whitney U tests as appropriate based on data skew. Sensitivity, specificity, and receiver operating curves (ROCs) were determined for group classification of PD-decline using LIBRA, MoPaRDS, and pPREDICT scores. ROC comparisons were made to test the differences in ROC area under the curves (AUCs) for LIBRA, MoPaRDS, and pPREDICT using chi-square difference test. Linear regression models were built with CCS as the continuous outcome, first with total LIBRA, MoPaRDS, or pPREDICT score as the predictors, then also with each of the individual risk factors as predictors. Statistical analyses were performed using the Stata 15 software (StataCorp. 2017. Stata Statistical Software: Release 15. College Station, TX: StataCorp LLC).

## 3. Results

Table 2 displays the characteristics of the *de novo* PPMI participants with PD included in the analysis. The PD-decline group (*n* = 44) was older (64.8 ± 9.1 versus 60.1 ± 9.8 years, *p* = 0.004) and with higher Unified Parkinson’s Disease Rating Scale (UPDRS) Pt. III motor score (22.4 ± 9.7 versus 19.5 ± 8.2, *p* = 0.04), Rapid Eye Movement (REM) Sleep Behavior Disorder Questionnaire (RSBDQ) (4.1 ± 3.0 versus 2.9 ± 2.4, *p* = 0.004), and Epworth Sleepiness Scale (ESS) (7.0 ± 3.8 versus 5.5 ± 3.1, *p* = 0.0003) scores compared to the PD-maintain group (*n* = 263). Additionally, the PD-decline group had higher LIBRA (6.76 ± 3.77, *p* = 0.0013), MoPaRDS (2.45 ± 1.41, *p* = 0.0001), and pPREDICT scores (4.52 ± 1.66, *p* < 0.001) compared to the PD-maintain group (LIBRA 4.98 ± 3.30, MoPaRDS 1.67 ± 1.16, pPREDICT 3.38 ± 1.69).

**Table 2:** PPMI participant baseline demographics and clinical information.

	All (*N* = 307)	PD-decline (*N* = 44)	PD-maintain (*N* = 263)	Value of *p*
Age (years)	60.8 ± 9.8	64.8 ± 9.1	60.1 ± 9.8	**0.004**
Sex (male)	66.1%	75.0%	64.6%	0.18
Race/ethnicity (non-white/non-Hispanic)	8.5%	13.6%	7.6%	0.18
Education (years)	15.7 ± 2.9	16.1 ± 3.3	15.6 ± 2.8	0.30
Screening MoCA*	27 ± 2.3	27 ± 2.3	27 ± 2.3	0.19
Cognition: CCS	0.13 ± 0.6	0.16 ± 0.7	0.12 ± 0.6	0.70
Obesity: BMI	27.1 ± 4.5	28.0 ± 4.1	26.9 ± 4.6	0.13
Symptom duration (months)	61.7 ± 56.7	63.4 ± 41.7	61.4 ± 58.9	0.85
Motor: UPDRS Pt. III	20.0 ± 8.5	22.4 ± 9.7	19.5 ± 8.2	**0.04**
REM sleep behavior disorder: RSBDQ	3.1 ± 2.5	4.1 ± 3.0	2.9 ± 2.4	**0.004**
Physical activity: PASE^ψ^	178.7 ± 105.4	176.4 ± 103.6	179.1 ± 105.8	0.87
Depression: GDS	5.2 ± 1.5	5.3 ± 1.5	5.2 ± 1.5	0.87
Anxiety: STAI-S	47.2 ± 5.4	46.8 ± 5.8	47.3 ± 5.3	0.64
Daytime sleepiness: ESS	5.7 ± 3.2	7.0 ± 3.8	5.5 ± 3.1	**0.003**
LIBRA		6.76 ± 3.77	4.98 ± 3.30	**0.0013**
MoPaRDS		2.45 ± 1.41	1.67 ± 1.16	**0.0001**
pPREDICT		4.52 ± 1.66	3.38 ± 1.69	**<0.001**

Data presented as mean ± standard deviation unless otherwise noted. *MoCA score collected at screening visit. ^ψ^PASE added to PPMI de novo cohort after trial start, therefore earliest available value for each participant used.

BMI, body mass index; CCS, composite cognitive score; ESS, Epworth Sleepiness Scale; GDS, Geriatric Depression Scale; LIBRA, Lifestyle for Brain Health; MoCA, Montreal Cognitive Assessment; PASE, Physical Activity Scale for the Elderly; PPMI, Parkinson’s Progression Markers Initiative; pPREDICT, preliminary Parkinson’s disease Risk Estimator for Decline In Cognition Tool; UPDRS, Unified Parkinson’s Disease Rating Scale; RSBDQ, REM Sleep Behavior Disorder Questionnaire; STAI-S, State–Trait Anxiety Inventory (State). Bolded items reached significance of *p*<0.05.

**Figure 1:**
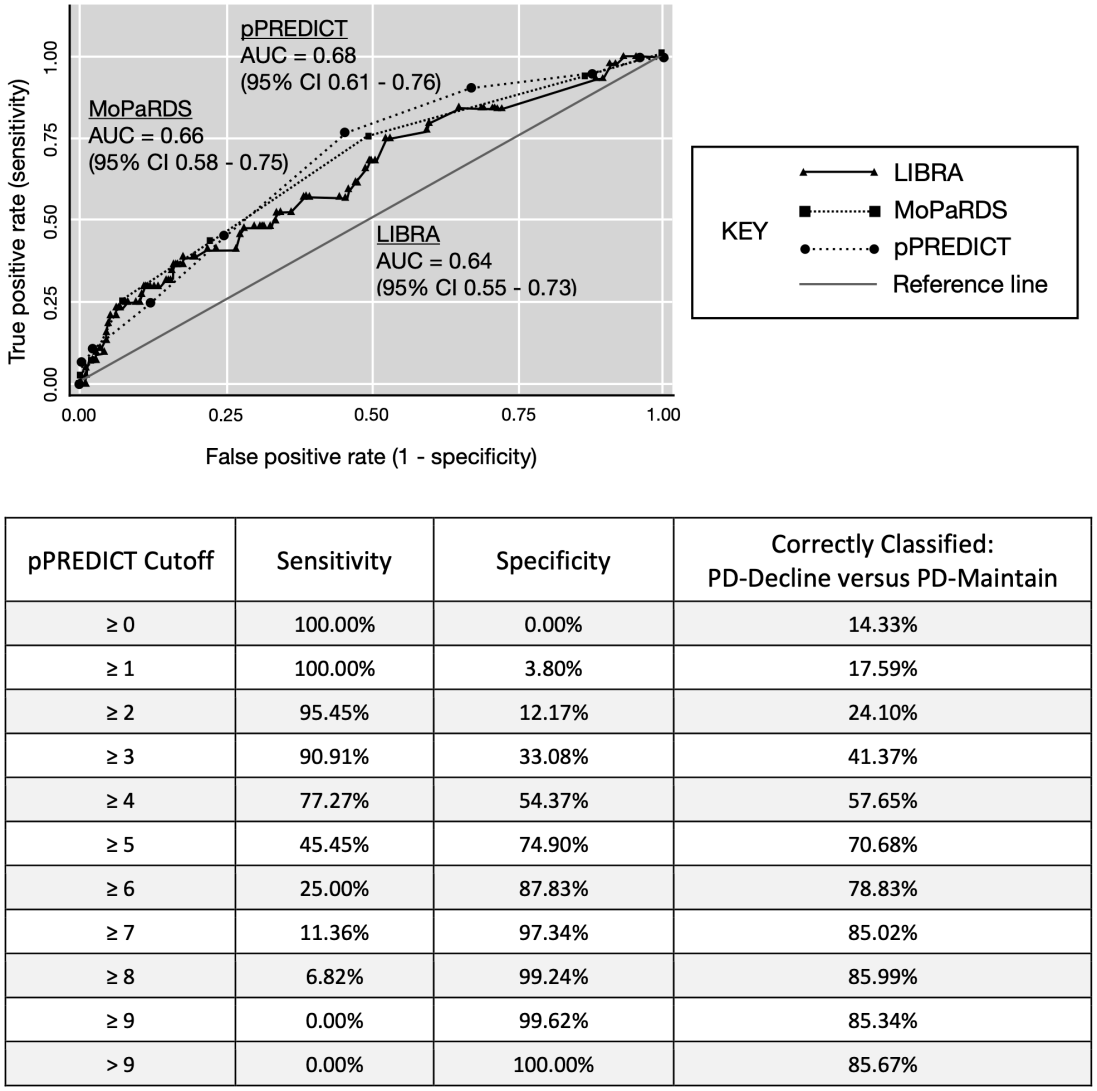
ROC-AUC curve for LIBRA and pPREDICT. AUC, area under the curve; LIBRA, Lifestyle for Brain Health; pPREDICT, preliminary Parkinson’s disease Risk Estimator for Decline In Cognition Tool. MoPaRDS, Montreal Parkinson Risk of Dementia Scale.

The ROC-AUC for distinguishing PD-decline versus PD-maintain was 0.64 (95% CI, 0.55–0.73) for LIBRA, 0.66 (95% CI, 0.58–0.75) for MoPaRDS, and 0.68 (95% CI, 0.61–0.76) for pPREDICT (Figure 1A). LIBRA score ≥ 4.2, chosen based on previous cutoff value for high risk (Vos et al., 2017), conferred 68.2% sensitivity and 49.4% specificity for identifying PD-decline which correctly classified 52.1% with a positive likelihood ratio (LR+) of 1.35 and negative LR (LR-) of 0.64. MoPaRDS score ≥ 4, chosen based on previous cutoff value (Dawson et al., 2018), conferred 25.0% sensitivity and 92.8% specificity for distinguishing PD-decline from PD-maintain which correctly classified 83.1% with a LR+ of 3.46 and LR- of 0.81. A pPREDICT cutoff of ≥4, chosen to maximize sensitivity without compromising sensitivity (Figure 1B), conferred 77.2% sensitivity and 54.3% specificity for identifying PD-decline which correctly classified 57.7% with a LR+ of 1.69 and LR- of 0.42. Comparing the ROC-AUC for LIBRA, MoPaRDS, and pPREDICT revealed no significant differences (chi-squared = 0.95, *p* = 0.62).

In linear regression analyses with change in CCS as the continuous dependent variable, total LIBRA (*n* = 307, *p* = 0.020), MoPaRDS (*n* = 307, *p* < 0.001), and pPREDICT (*n* = 305, *p* < 0.001) scores were associated with change in CCS. In a linear regression analysis with the nine LIBRA risk factors that were included as independent variables, the R-squared value was 0.063. The LIBRA risk factor that contributed to the model was age stratified by sex (coefficient −0.044, 95% CI -0.070 – −0.019; *p* = 0.001; Table 3). In a linear regression analysis with the eight MoPaRDS risk factors that were included as independent variables, the R-squared value was 0.13. For MoPaRDS, age older than 70 years (coefficient − 0.26, 95% CI -0.41 – −0.099; *p* = 0.001) and report of hallucinations (coefficient −0.90, 95% CI -1.3 – −0.53; *p* < 0.001) contributed to the model (Table 3). In a linear regression analysis with the fourteen pPREDICT risk factors as independent variables, the R-squared value was 0.15. For pPREDICT, male sex (coefficient 0.15, 95% CI 0.0028–0.30; *p* = 0.046), older age (coefficient −0.25, 95% CI −0.39 – −0.12; *p* < 0.001), excessive daytime sleepiness (coefficient −0.47, 95% CI −0.71 – −0.23; *p* < 0.001), and moderate–severe motor symptoms (coefficient −0.30, 95% CI −0.52 – −0.072; *p* = 0.010) contributed to the model (Table 3).

**Table 3:** Linear regression analyses of the association between baseline risk factors for LIBRA and pPREDICT with change in CCS over 5 years.

	Coefficient (95% CI)	*p* value
*LIBRA*		
Age by sex	−0.044 (−0.070 – −0.019)	**0.001**
Education level	0.023 (−0.10–0.15)	0.71
Depression (GDS-15 ≥ 5)	−0.033 (−0.10–0.039)	0.37
Hypertension	−0.041 (−0.13–0.050)	0.38
Hyperlipidemia	−0.022 (−0.13–0.087)	0.69
Diabetes	0.17 (−0.061–0.41)	0.15
Coronary artery disease	−0.13 (−0.37–0.11)	0.30
Obesity (BMI ≥30 kg/m^2^)	−0.031 (−0.12–0.061)	0.50
Physical inactivity (PASE ≤90)	−0.088 (−0.24–0.067)	0.26
*MoPaRDS*		
Male sex	−0.12 (−0.26–0.015)	0.081
≥70 years old	−0.26 (−0.41 – −0.099)	**0.001**
Mild cognitive impairment (MoCA <26)	−0.14 (−0.29–0.015)	0.076
Bilateral disease onset	−0.019 (−0.19–0.16)	0.83
REM sleep behavior disorder (RSBDQ >5)	−0.088 (−0.26–0.080)	0.30
Hallucinations	−0.90 (−1.3 – −0.53)	**< 0.001**
Falls or freezing	−0.10 (−0.36–0.16)	0.44
Orthostatic SBP drop >10 mmHg	−0.052 (−0.20–0.097)	0.49
*pPREDICT*		
Male sex	0.15 (0.0028–0.30)	**0.046**
≥65 years old	−0.25 (−0.39 – −0.12)	**< 0.001**
≥5 year disease duration*	0.067 (−0.066–0.20)	0.32
Non-white and/or Hispanic	−0.23 (−0.46 – −0.00019)	0.050
≤12 years education	0.051 (−0.13–0.23)	0.57
Screening MoCA <26	−0.083 (−0.24–0.077)	0.31
Depression (GDS-15 ≥ 8)	−0.012 (−0.19–0.17)	0.90
Anxiety (STAI-S ≥ 54)*	−0.042 (−0.29–0.20)	0.73
Excessive Daytime Sleepiness (ESS ≥11)*	−0.47 (−0.71 – −0.23)	**< 0.001**
REM sleep behavior disorder (RSBDQ ≥ 5)*	0.0012 (−0.17–0.18)	0.99
Moderate/severe motor symptoms (UPDRS Pt. III ≥ 32)*	−0.30 (−0.52 – −0.072)	**0.01**
≥2 vascular risk factors	0.020 (−0.14–0.18)	0.80
Obesity (BMI ≥25 kg/m^2^)	0.0061 (−0.13–0.15)	0.93
Sedentary/light physical activity (PASE ≤90)	−0.040 (−0.21–0.13)	0.64

^*^Risk factors that are unique to pPREDICT when compared to LIBRA. Italicized risk factors are PD-specific.

BMI, body mass index; ESS, Epworth Sleepiness Scale; GDS, Geriatric Depression Scale; LIBRA, Lifestyle for Brain Health; MoCA, Montreal Cognitive Assessment; PASE, Physical Activity Scale for the Elderly; pPREDICT, preliminary Parkinson’s disease Risk Estimator for Decline In Cognition Tool; UPDRS, Unified Parkinson’s Disease Rating Scale; RSBDQ, REM Sleep Behavior Disorder (RSBD) Questionnaire; SBP, systolic blood pressure; STAI-S, State–Trait Anxiety Inventory (State). Bolded items reached significance of *p*<0.05.

## 4. Conclusion

There is currently no disease-modifying therapy available for cognitive impairment in PD (Sun and Armstrong, 2021). Potentially modifiable risk factors are associated with 40% of all-cause dementia cases worldwide (Livingston et al., 2020); therefore, lifestyle modifications may potentially impact cognitive outcomes in PD. For example, there is evidence that aerobic exercise improves cognition in PD-MCI (Sun and Armstrong, 2021). The goal of this study was to adapt or explore development of a PD-specific predictor tool focusing on modifiable and/or treatable risk factors while being user-friendly and easily applied in a variety of clinical settings. Given that many individuals with PD do not receive care from movement disorder specialists – and even often do not have regular appointments with a neurologist (Pearson et al., 2023) – the development of a simple tool to help guide personalize discussions about cognitive decline risk is of significant importance. Such a predictor tool would provide personalized recommendations for specific individuals with PD rather than relying on general recommendations for the masses.

LIBRA was an ideal starting point as a tool developed for all-cause dementia focusing on modifiable lifestyle factors (Deckers et al., 2015). LIBRA was associated with change in CCS over 5 years in this PD population. Importantly, the only LIBRA risk factor that contributed to the model was non-modifiable age stratified by sex. Of note, LIBRA scoring applies higher risk to female sex for every age > 65 years, whereas male sex has higher risk of cognitive decline in PD. MoPaRDS is a PD-specific tool developed for use in clinical settings (Dawson et al., 2018; Reekes et al., 2020), therefore is also an important tool for comparison given that it holds promise for scalability to a wider PD population. MoPaRDS was also associated with change in CCS over 5 years; however, only non-modifiable older age and report of hallucinations contributed to the linear regression model. The presence of hallucinations alone is associated with an increased risk of mortality in individuals with PD (Isaacson and Citrome, 2022). Hallucinations are a treatable symptom in PD, however antipsychotics can worsen motor symptoms and increase the mortality in individuals with dementia. Therefore, unmodified LIBRA and MoPaRDS risk tools may both identify at-risk individuals with PD, but neither highlight actionable lifestyle modifications or treatable symptoms to potentially mitigate this risk.

pPREDICT may provide more PD-specific information on the risk of cognitive decline compared to LIBRA while also focusing on potentially modifiable risk factors unlike the PD-specific MoPaRDS. Although the ROC-AUC were not significantly different between LIBRA, MoPaRDS, and pPREDICT, pPREDICT has the potential for being more PD-specific while also focusing on potentially modifiable riss and/or treatable symptoms. Not only was the total pPREDICT score associated with change in CCS from baseline to 5-year follow-up in this PD population, but multiple individual risk factors contributed including male sex, older age, excessive daytime sleepiness, and moderate–severe motor symptoms. Therefore, potentially treatable risk factors contributed to the pPREDICT model, which could represent actionable targets for personalized risk reduction.

There are multiple advantages to pPREDICT. It uses demographic and clinical information that can be collected without additional invasive and/or expensive diagnostic testing. It also does not rely on specific neuropsychological tests rather uses a cognitive screen (i.e., MoCA) that can be performed in a variety of clinical settings where individuals with PD regularly receive care. Therefore, while the performance of previously reported cognitive decline predictor tools are superior in their respective cohorts (Schrag et al., 2017; Dawson et al., 2018; Gramotnev et al., 2019), pPREDICT holds potential for wider applicability outside of tertiary/quaternary care centers or in the research setting. Unlike other office-based tools for predicting PD-associated cognitive decline including the MoPaRDS, it focuses on potentially reversible/modifiable risk factors. Additionally, the simplicity of using dichotomized risk factors may allow for application to clinical settings that use alternative questionnaires. For example, although PPMI used the Geriatric Depression Scale Short Form (GDS-15) for depression assessment, there are other depression scales with validated cutoff values in PD (i.e., Hospital Anxiety and Depression Scale (Mondolo et al., 2006)) that could potentially be used with pPREDICT; however, pPREDICT needs to be validated in cohorts using alternative scales to determine applicability outside of the PPMI-chosen questionnaires.

Excessive daytime sleepiness was significantly associated with cognitive decline in the pPREDICT model. Excessive daytime sleepiness has been suggested as a marker of more severe disease in PD, however there are also potential treatable causes of excessive daytime sleepiness in individuals with PD including unrecognized/undiagnosed sleep apnea or more broadly sleep disordered breathing, insomnia, or uncontrolled restless leg syndrome symptoms (Zuzuarregui and During, 2020). Excessive daytime sleepiness can be treated with light therapy (Zuzuarregui and During, 2020), although meta-analysis has not shown benefit for excessive daytime sleepiness, depressive symptoms, or motor symptoms (Huang et al., 2021). Additionally, the potential longer-term benefits on cognitive outcomes of treating excessive daytime sleepiness in PD are currently unknown, however an area of interest given that it may be a potentially modifiable risk. At this time, it is not clear whether excessive daytime sleepiness is a sign of more severe or progressive disease – therefore associated with cognitive impairment – but given that excessive daytime sleepiness may have potential treatable causes with cognitive impacts, it is important to be aware of the potential role in individualized risk reduction.

Moderate to severe motor symptoms was also significantly associated with cognitive decline in the pPREDICT model. Although this association is likely to be multifactorial including a relationship with disease severity, a potential modifiable/treatable mechanism is physical activity. A questionnaire to assess for physical activity (i.e., the Physical Activity Scale for the Elderly [PASE]) was a measurement added to PPMI after the trial started (Mantri et al., 2018), therefore values for scoring pPREDICT were not limited to baseline assessments for all the participants creating some variability. Additionally, there is evidence of individuals with PD over-reporting activity as compared to objective measurements (Mantri et al., 2019). Despite the hurdles of studying physical activity in large cohort studies, there is evidence supporting physical activity for improved motor outcomes (Ernst et al., 2023), as well as evidence for exercise improving cognition outcomes in PD (Sun and Armstrong, 2021). There may be variable impact on specific cognitive domains with differential responses for specific modalities (Sun and Armstrong, 2021), however the longer-term benefits on cognitive outcomes is an area of active research. Physical activity is a modifiable risk for all-cause dementia (Livingston et al., 2020), therefore is important to highlight in individuals with PD especially with increased risk of cognitive and motor decline.

The other potentially modifiable and/or treatable risk factors in pPREDICT that did not significantly contribute to the model included REM sleep behavior disorder (RSBD), depression, anxiety, obesity, and multiple vascular risk factors. One potential reason for RSBD not significantly contributing to the model for MoPaRDS or pPREDICT was using clinical history via the RSBDQ rather than confirmed diagnosis by polysomnography. REM sleep behavior disorder in PD is associated with worse cognitive performance (Maggi et al., 2021), therefore there is interest in targeting RSBD for improvement of cognitive outcomes in PD and in idiopathic cases. Depression and anxiety are both common non-motor symptoms associated with PD, however the screens (i.e., GDS-15, State-Trait Anxiety Inventory [STAI]) may be capturing other symptoms in PD such as apathy and fatigue (i.e., GDS-15) (Lopez et al., 2018) and autonomic dysfunction (i.e., STAI) (Wang et al., 2023). As a result, it is possible these screens are not sensitive enough for depression or anxiety to provide the necessary signal for pPREDICT. Higher body mass index (BMI) at baseline has previously been shown to be associated with cognitive decline in the PPMI cohort per sequential MoCA assessments (Forbes et al., 2021), however higher BMI did not significantly contribute to the pPREDICT model perhaps as a result of using CCS as the outcome. Additionally, vascular risk factors in the setting of PD are associated with performance on specific cognitive domains (Pilotto et al., 2016), therefore using CCS may have masked any contributions to the pPREDICT model.

The dataset and approach used to develop pPREDICT have several limitations. The PPMI cohort is highly educated compared to the general population as is not uncommon for clinical trials, so it needs to be validated in other cohorts including those with lower educational status to determine the applicability to other populations. Additionally, cognitive decline in PD takes years to develop, so the relatively short follow-up of 5 years may be too short. The predicted conversion rate from PD-MCI to PDD is about 10% per year (Sun and Armstrong, 2021) and nearly half of individuals with PD and normal cognition develop cognitive impairment within 6 years (Pigott et al., 2015). Additionally, the follow-up duration for pPREDICT is longer (Schrag et al., 2017; Hogue et al., 2018; Yousaf et al., 2019; Wilson et al., 2020) or on par (Dawson et al., 2018; Gramotnev et al., 2019) with prior cognitive decline predictor tools. This initial pPREDICT does not correct for absent neuropsychological data at 5-year follow-up, which may be concentrated for individuals experiencing decline thereby producing bias in the data. A future approach to correct for missingness by imputing data based on the trajectory of available follow-up will clarify whether the model improves. Lastly, using change in CCS as the outcome measure of cognitive decline may mask decline isolated to specific cognitive domains that may be more sensitive to PD-specific cognitive changes. Investigating the risk factors associated with decline in specific neuropsychological tests will be informative in the future.

This is a first step in developing a pragmatic and practical tool to predict cognitive decline in PD focusing on potentially modifiable risk factors. Further refinement of the tool aimed at improving performance while maintaining simplicity is the next step. Once a refined tool is developed, external validation will be important to determine wider applicability and scalability. In the future, this tool may allow for personalized counseling on actionable lifestyle modifications and other disease-specific treatments to reduce risk of cognitive decline in individuals with PD.

## Data availability statement

Publicly available datasets were analyzed in this study. This data can be found here: https://www.ppmi-info.org/.

## Ethics statement

The studies involving humans were approved by Colorado Multiple Institutional Review Board. The studies were conducted in accordance with the local legislation and institutional requirements. Written informed consent for participation was not required from the participants or the participants’ legal guardians/next of kin in accordance with the national legislation and institutional requirements.

## Author contributions

TC: Conceptualization, Data curation, Funding acquisition, Investigation, Methodology, Project administration, Writing – original draft, Writing – review & editing. LM: Methodology, Supervision, Writing – review & editing. SH: Conceptualization, Formal analysis, Methodology, Supervision, Writing – review & editing.

## Footnotes

^1^www.ppmi-info.org/access-data-specimens/download-data
